# Auswirkungen der Coronapandemie auf soziale Netzwerke in Risikofamilien

**DOI:** 10.1007/s00278-021-00491-9

**Published:** 2021-02-10

**Authors:** André Knabe, Michael Kölch, Carsten Spitzer, Olaf Reis

**Affiliations:** 1 Institut für Soziologie und Demographie, Universität Rostock, Ulmenstr. 69, 18057 Rostock, Deutschland; 2 Klinik für Psychiatrie, Neurologie, Psychosomatik und Psychotherapie im Kindes- und Jugendalter, Universitätsmedizin Rostock, Rostock, Deutschland; 3 Klinik für Psychosomatische Medizin und Psychotherapie, Universitätsmedizin Rostock, Rostock, Deutschland

**Keywords:** Coping-Verhalten, COVID-19, Familie, Soziales Unterstützungssystem, „Mixed methods“, Coping behavior, COVID-19, Family, Social support system, Mixed methods

## Abstract

**Hintergrund:**

Die Maßnahmen zur Reduktion des Infektionsgeschehens im Rahmen der Coronapandemie brachten insbesondere für Familien mit schulpflichtigen Kindern erhebliche Einschränkungen mit sich. Besonders betroffen sind Risikofamilien, die bereits vor der Pandemie mit psychischen Störungen, Armut oder beengtem Wohnraum konfrontiert waren.

**Fragestellung:**

Wie stellen sich Zusammensetzung und Dynamik der sozialen Netzwerke von Risikofamilien während des Lockdowns dar?

**Material und Methode:**

„Mixed-methods“-Analyse auf Basis von 19 qualitativen Leitfadeninterviews und 18 standardisiert erhobenen egozentrierten Netzwerken mit 224 von den Befragten („ego“) genannten Personen („alter“).

**Ergebnisse:**

Netzwerke werden durch die Krise geschwächt. Gleichzeitig sind sie wichtigste Ressource der Bewältigung. Unterstützung fehlt v. a. bei der Sorge um die psychisch erkrankten Kinder. Wichtigste Quellen von Unterstützung sind die erweiterte Familie und institutionelle Einrichtungen. Letztere waren in der Krise oft nur noch eingeschränkt oder gar nicht mehr zu erreichen. Im Idealfall sind die verbliebenen Beziehungen stark genug, um die Situation zu überstehen. Im schlimmsten Fall werden die Kinder nicht nur aus institutionellen Strukturen entlassen (Kita, Schule, medizinische und therapeutische Hilfen), sondern verschwinden ganz und gar aus der institutionellen und gesellschaftlichen Obhut. Ihr Wohl hängt dann allein von den Kompetenzen ihrer Eltern ab.

**Schlussfolgerung:**

In Zeiten von Kontaktbeschränkungen benötigen Risikofamilien besondere Aufmerksamkeit. Es genügt nicht, darauf zu warten, dass sie von sich aus um Hilfe oder eine Notbetreuung für die Kinder bitten. Institutionen sollten proaktiv praktische und informationelle Unterstützung anbieten.

## Abstract (en)

**Background:**

The measures taken to reduce the incidence of infections during the corona pandemic brought about considerable restrictions, especially for families with school-age children. Particularly affected are families at risk, who were already confronted with mental disorders, poverty and cramped housing before the pandemic.

**Research question:**

How does the lockdown affect the composition and dynamics of the social networks of risk families?

**Material and method:**

Mixed methods analysis based on 19 problem-centered interviews and 18 standardized egocentered networks with 224 persons (alter) named by the interviewees (ego).

**Results:**

Networks are weakened by the crisis. At the same time, they are the most important resource for coping. Support is lacking, especially in the care of mentally ill children. The most important sources of support are the extended family and institutional facilities. The latter proved to be particularly unreliable during the crisis. In the best case the remaining relationships are strong enough to survive the situation. In the worst case the children are not only discharged from institutional structures (day care, school, medical and therapeutic assistance) but disappear completely from institutional and social custody. Their well-being then depends solely on the competence of their parents.

**Conclusion:**

In times of contact restrictions, families at risk need special attention. It is not enough to wait for them to ask for help or emergency care for their children. Institutions should proactively offer practical and informal support.

Die Coronakrise belastet Familien v. a. durch den Wegfall von Beziehungen. Der damit einhergehende Verlust alltäglicher Strukturen und von Unterstützung erzeugt einen Kompensationszwang, der auf der Kernfamilie lastet. In dieser Situation sind Risikofamilien mit psychischen Problemen und einer hohen Abhängigkeit von institutionellen Hilfen besonders bedroht. In der vorgestellten Studie wurde der Frage nachgegangen, wie sich Zusammensetzung und Dynamik der sozialen Netzwerke von Risikofamilien während des Lockdowns darstellen.

## Einführung

Die infolge der Coronapandemie durchgesetzten Maßnahmen zur Reduktion des Infektionsgeschehens bedeuteten insbesondere für Familien mit schulpflichtigen Kindern erhebliche Einschränkungen (Müller et al. 2020). Homeschooling, Homeoffice und Kontaktsperren brachten alltägliche Handlungspraxen zum Einsturz und forderten das Bewältigungshandeln heraus. Bislang in der Literatur diskutierte Auswirkungen der Krise sind: unter Druck geratene Geschlechterarrangements (Craig 2020; Czymara et al. 2020; Hank und Steinbach 2020,), die Zunahme häuslicher Gewalt (Amarel et al. 2020), Kindeswohlgefährdung (Jentsch und Schnock 2020; Klitzing 2020), psychosozialer Stress (Marroquín et al. 2020) und die Abhängigkeit von sozialer Unterstützung (Koos und Bertogg 2020; Pitas und Ehmer 2020). Doch nicht alle Familien sind gleichermaßen von der Krise betroffen, und es ist anzunehmen, dass sich bestehende Ungleichheiten in der Krise verschärfen (Andresen et al. 2020). Die vorliegende Untersuchung richtete daher den Blick auf Risikofamilien, die bereits vor der Pandemie mit psychischen Störungen, Armut oder beengtem Wohnraum konfrontiert waren.

Die Sozialpsychologie und die soziologische Netzwerkforschung gehen davon aus, dass die Bewältigung solcher Krisen nicht nur auf Basis kognitiver, materieller und kultureller Ressourcen erfolgt, sondern in entscheidendem Maße durch die soziale Einbettung der Akteur*innen in soziale Beziehungsstrukturen beeinflusst wird (Granovetter 1985; Stegbauer und Clemens 2020). Allerdings beeinflussen die Maßnahmen zur Eindämmung der Pandemie nicht nur die Situation der Familien selbst, sondern auch ihre Netzwerke. Der Lockdown wirft das Bewältigungshandeln auf die Mitglieder des eigenen Haushalts zurück (Reis 2020). Während einige wenige Beziehungen in den Kernnetzwerken in besonderem Maße gefordert waren, war der Zugang zu darüber hinausgehenden sozialen Kreisen begrenzt oder ganz unterbrochen. In der vorgestellten Studie wurde daher gefragt, wie sich Zusammensetzung und Dynamik der sozialen Netzwerke von Risikofamilien während des Lockdowns darstellen.

## Daten und Methode

Der Datensatz setzt sich aus 19 problemzentrierten Interviews zusammen, die im Mai und im Juni 2020 geführt wurden, also unmittelbar nach den ersten Lockerungen der strengsten Coronamaßnahmen in der ersten Welle. Die meisten öffentlichen Orte waren noch gesperrt, doch Angehörige „systemrelevanter“ Berufsgruppen durften ihre Kinder wieder zur Schule und in die Kita schicken, und die Ausgangssperren wurden gelockert (Beitrag von Reis et al., im selben Heft).

Interviewt wurden Eltern aus 15 Familien, in denen eine diagnostizierte psychische Störung vorlag, und aus 4 Familien ohne psychiatrische Diagnose. Am Ende des Gesprächs wurden die sozialen Beziehungen der Befragten mithilfe der folgenden 5 Namensgeneratoren erhoben:

1. Mit welchen Personen oder Institutionen waren Sie zur Zeit der Ausgangsbeschränkungen in der Coronakrise noch in Kontakt?
2. Gibt es Personen oder Institutionen, die Sie aufgrund der Ausgangsbeschränkungen und Kontaktverbote nicht mehr treffen konnten?
3. Haben Sie in dieser Zeit Kontakte zu neuen Personen oder Institutionen geknüpft?
4. Wer hat Sie der letzten Zeit seit Ausbruch der Coronakrise unterstützt?
5. Gab es Personen oder Institutionen, für die Sie seitdem Unterstützung geleistet haben?

Insgesamt wurden auf diese Weise 18 Netzwerke mit einer Gesamtzahl von 224 Personen („alteri“) erhoben^1^. Bei vielen der 224 Alteri handelt es sich um multiplexe Beziehungen, d. h., sie wurden mehrfach genannt, z. B. wenn eine Befragte mit einem Freund in Kontakt war (Nennung bei der 1. Frage), der Unterstützung geleistet (4. Frage), aber auch von ihr empfangen hat (5. Frage). Nach der Nennung der Namen wurden die Befragten gebeten anzugeben, welche der genannten Personen miteinander bekannt sind („alter-alter ties“). Auf Basis dieser Angaben wurden Visualisierungen der Netzwerke erstellt, die gemeinsam mit den transkribierten Interviews zur Analyse der Wahrnehmung und Bewältigung der Situation im Kontext der sozialen Einbettung herangezogen wurden (in Anlehnung an die qualitative strukturale Analyse nach Herz et al. 2015).

## Soziale Netzwerke von Familien in der Coronakrise

Im ersten Teil des Beitrags werden Ergebnisse der quantitativen Inspektion der Strukturen und Dynamiken der Familiennetzwerke vorgestellt. Im zweiten Teil werden die Wechselwirkungen zwischen den Netzwerken und dem Bewältigungshandeln anhand von 3 Fallskizzen aufgezeigt.

### Netzwerkstrukturen und -dynamiken in Risikofamilien

#### Zusammensetzung

Die Verteilung von Netzwerkmaßen im Sample zeigt Tab. 1. Die Netzwerke haben eine mittlere Größe von 12 Alteri. Das kleinste besteht aus 7 Personen, und es gibt auch einige sehr große Netzwerke mit mehr als 16 Personen („cut off“, 3. Quartil = 16).

**Table 1:** 

	Min.	1. Quartil	Median	Mittelwert	3. Quartil	Max.
Größe	7	9	12	12,44	16	19
Dichte	0,06	0,18	0,32	0,38	0,54	0,95
Anteil der Kernfamilie	0	0,11	0,18	0,18	0,24	0,43
Anteil der erweiterten Familie	0	0,15	0,25	0,24	0,32	0,40
Anteil der Freunde, Bekannten und Kollegen	0,07	0,18	0,25	0,28	0,36	0,63
Anteil der Institution	0	0,11	0,23	0,26	0,38	0,58

**Figure 1:**
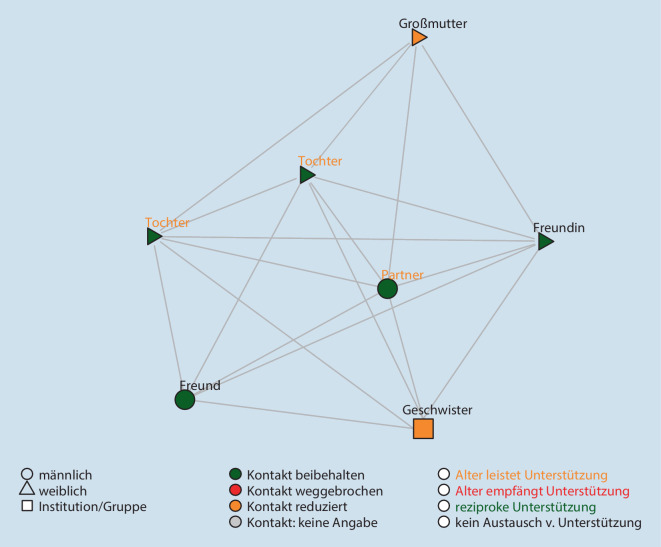

**Figure 2:**
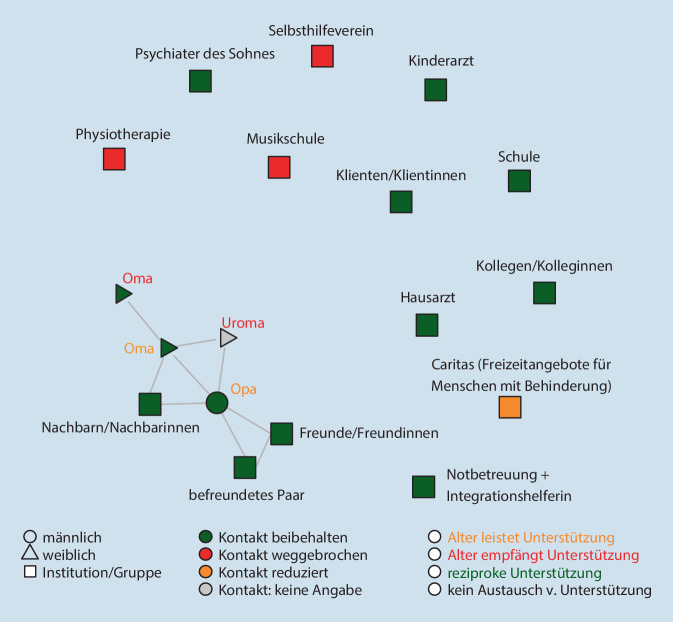

Die Netzwerkdichte gibt Auskunft über den Anteil der bestehenden Beziehungen an allen möglichen Beziehungen. Was das bedeutet, zeigt sich gut in der Visualisierung: Besonders hoch ist die Dichte des Netzwerks in Abb. 1, besonders gering in Abb. 2. Im Mittel existieren 38 % aller möglichen Beziehungen in den Netzwerken.

Die übrigen 4 Parameter beschreiben Netzwerkzusammensetzungen. Den durchschnittlich größten Anteil aller Beziehungen machen Freund*innen, Bekannte und Kolleg*innen (28 %) aus, dicht gefolgt vom Anteil der Beziehungen zu Institutionen (26 %). Rechnet man jedoch den Anteil der Kernfamilie (18 %) und den der erweiterten Familienmitglieder (24 %) zusammen, zeigt sich ein deutliches Übergewicht familiärer Alteri (42 %).

#### Dynamik

In Tab. 2 ist aufgeführt, welche der Beziehungen während des Lockdowns beibehalten, reduziert oder abgebrochen wurden, und welche neu hinzugekommen sind. Folgende Kategorien wurden auf Basis der standardisierten Netzwerkabfrage (Namensgeneratoren; s. Abschn. „Daten und Methode“) gebildet:

- *Kontakt beibehalten*: Nennung auf die 1. Frage,
- *Kontakt abgebrochen*: Nennung auf die 2. Frage,
- *Kontakt reduziert*: Nennung auf die 1. und 2. Frage,
- *neue Kontakte*: Nennung auf die 3. Frage.

**Table 2:** 

	Min.	1. Quartil	Median	Mittelwert	3. Quartil	Max.
Kontakt beibehalten	0,25	0,64	0,74	0,73	0,84	1,00
Kontaktreduktion	0	0	0,08	0,13	0,22	0,58
Kontaktabbruch	0	0	0,06	0,08	0,16	0,26
Neue Kontakte	0	0	0	0,03	0,05	0,17

Es zeigt sich, dass die Beziehungen zu etwa drei Viertel (74 %) der Personen im Netzwerk weiterbestehen; die übrigen Beziehungen wurden eingeschränkt (13 %) oder ganz abgebrochen (8 %)^2^. Das Ausmaß der Kontaktreduktion unterscheidet sich in den Netzwerken erheblich. Im obersten Quartil sind 22–58 % der Beziehungen reduziert bzw. 16–26 % der Beziehungen ganz weggebrochen, während sich im untersten Quartil gar keine Reduktionen und Abbrüche finden. Neue Kontakte wurden dagegen kaum geknüpft, der Mittelwert beträgt 3 %, mindestens die Hälfte der Befragten hat gar keinen neuen Kontakt hinzugewonnen (Median = 0).

#### Beziehungen sind unterschiedlich betroffen

Deutliche Unterschiede zeigen sich, wenn gefragt wird, welche Beziehungen besonders beeinträchtigt waren (Tab. 3). In der Kernfamilie bleiben fast alle Beziehungen erhalten (97 %). In allen anderen Domänen gibt es Einschnitte. Die Beziehungen zur erweiterten Familie wurden stark reduziert (29 %), aber nur sehr selten komplett abgebrochen (4 %). Insbesondere der Kontakt zu Großeltern wurde aus Sorge vor Ansteckung häufig nur noch telefonisch und nicht mehr persönlich geführt. Die Kontakte zu Freund*innen, Kolleg*innen und Bekannten wurden in geringerem Ausmaß reduziert, während Kontakte zu Institutionen eher abgebrochen als reduziert wurden. Dies führt in vielen Fällen zu einem spürbaren Verlust an sozialer, pädagogischer und gesundheitlicher Unterstützung im Alltag der Befragten und setzt die Bewältigung unter Druck (s. Fallbeispiele).

**Table 3:** 

	Kernfamilie		Erweiterte Familie		Freunde, Bekannte und Kollegen		Institution	
	Abs.	Rel.	Abs.	Rel.	Abs.	Rel.	Abs.	Rel.
Kontakt beibehalten	36	0,97	32	0,61	46	0,74	40	0,65
Kontaktreduktion	0	0	15	0,29	6	0,10	3	0,05
Kontaktabbruch	0	0	2	0,04	4	0,06	14	0,23
Nicht genannt	1	0,03	3	0,06	6	0,10	4	0,07
**Summe**	**37**	**1**	**52**	**1**	**62**	**1**	**61**	**1**

#### Beziehungen sind unterschiedlich relevant

Das Zurück-geworfen-Sein auf den eigenen Haushalt muss bewältigt werden. Eine wichtige Grundlage dieses Coping ist die Verfügbarkeit sozialer Unterstützung. Auch diese lässt sich auf Basis der in Abschn. „Daten und Methode“ aufgelisteten Namensgeneratoren kategorisieren:

- *Alter leistet Unterstützung*: Nennung auf die 4. Frage,
- *Alter empfängt Unterstützung*: Nennung auf die 5. Frage,
- *Reziproke Unterstützung*: Nennung auf die 4. und 5. Frage,
- *keine Unterstützung*: Nennung, weder auf die 4. noch auf die 5. Frage.

Die unterstützenden Beziehungen sind in Tab. 4 nach dem Beziehungstyp aufgeschlüsselt. Ein Drittel (32 %) aller Beziehungen in der Kernfamilie wurde als unterstützend gekennzeichnet, darunter sowohl Partner*innen als auch Kinder. Weitere Quellen sozialer Unterstützung sind die erweiterte Familie (21 %, häufig die Eltern der Befragten) und Institutionen (15 %). Freund*innen, Kolleg*innen und Bekannte spielen eine eher untergeordnete Rolle.

**Table 4:** 

	Kernfamilie		Erweiterte Familie		Freunde, Bekannte und Kollegen		Institution	
	Abs.	Rel.	Abs.	Rel.	Abs.	Rel.	Abs.	Rel.
Ego empfängt Unterstützung	12	0,32	11	0,21	4	0,065	9	0,15
Ego leistet Unterstützung	4	0,11	7	0,13	4	0,065	2	0,03
Reziproke Unterstützung	1	0,03	1	0,02	5	0,08	0	0
Keine Unterstützung	20	0,54	33	0,63	49	0,79	50	0,82
**Summe**	**37**	**1**	**52**	**1**	**62**	**1**	**61**	**1**

Der Blick auf die Gegenseite „keine Unterstützung“ offenbart einen hohen Wert von 82 % bei den Institutionen. Dieser lässt sich teilweise dadurch erklären, dass auch jene Institutionen enthalten sind, zu denen der Kontakt eingeschränkt (5 % aller Institutionen; Tab. 3) oder abgebrochen (23 %; Tab. 3) wurde.

Geleistet wird Unterstützung v. a. in der Kernfamilie (11 % + 3 % reziproke Unterstützung) und in der erweiterten Familie (13 % + 2 %). Die Unterstützung ist nur selten, und wenn doch, am ehesten im Freundes- und Bekanntenkreis reziprok.

### Netzwerk und Bewältigung: Fallbeispiele

Die Abb. 1, 2 und 3 zeigen die Netzwerke von 3 exemplarischen Familien^3^. Zu sehen sind alle Personen (Alteri), die im Zuge der standardisierten Netzwerkabfrage genannt wurden, sowie die durch Linien gekennzeichneten Beziehungen zwischen diesen Personen. Die Befragten selbst sind nicht in der Visualisierung enthalten. Es handelt sich bei den Darstellungen nicht um klassische egozentrierte Soziogramme (Moreno 1934), sondern um computergenerierte Visualisierungen der Netzwerkstruktur, in denen die Alteri auf Basis der Alter-Alter-Beziehungen angeordnet werden (Yousefi Nooraie et al. 2020). Die Analyse der subjektiven Bedeutung des Netzwerks für die Befragten erfolgte unter Rückgriff auf das qualitative Material. Farblich gekennzeichnet wurden die oben quantifizierten Angaben zur Beschreibung der Beziehungen während des Lockdowns.

#### Familie Fiedler^4^ (K04): „Familie ist uns sowieso heilig!“^5^

Das in Abb. 1 dargestellte Netzwerk der Familie Fiedler gehört zu den kleinsten im Sample (7 Alteri) mit einer besonders hohen Beziehungsdichte (0,9). Bis auf zwei Freund*innen besteht es ausschließlich aus familiären Beziehungen. Institutionelle Kontakte kommen nicht vor.

Die Fiedlers leben mit ihren Töchtern in einem kleinen Einfamilienhaus. Beide Eltern arbeiteten während des Lockdowns in Vollzeit weiter. Die 18-jährige Tochter absolviert eine schulische Ausbildung und ist derzeit im Homeoffice. Die 10-jährige Tochter war ebenfalls im Homeschooling, was jedoch mit der Aufnahme in eine psychiatrische Tagesklinik endete.

Die Eltern beschreiben sie als ein Kind, das sich permanent große Sorgen macht und dann „in sich fällt“ (K04§178), was in der pandemischen Situation häufiger auftrete, z. B. aus Sorge vor Ansteckung. Seit Beginn der Krise arbeitet Frau Fiedler nur noch in der Frühschicht; sie steht um 3:30 Uhr auf und beginnt um 5:00 Uhr. Herr Fiedler schläft vormittags und arbeitet in der Nacht. Wenn Frau Fiedler am frühen Nachmittag nach Hause kommt, bereitet sie ihren Töchtern ein Mittagessen zu und hilft der Kleinen bei den Schularbeiten.

Die Einschränkung sozialer Kontakte empfand sie insbesondere in Bezug auf ihre Mutter und die Geschwister als sehr einschneidend, da diese normalerweise ein fester Bestandteil des Familienlebens sind. In der Folge ist die Familie mit der Bewältigung allein. Herr und Frau Fiedler sind nicht nur im Beruf, sondern auch zu Hause voll gefordert und haben trotzdem „ein schlechtes Gewissen, dieses Kind den ganzen Tag allein zu lassen“ (K04§119).

Die Familie entwickelte nach und nach eine eigene Coronazeitstruktur, an der sich die 10-jährige Tochter in Abwesenheit der Eltern orientieren konnte. Sie bekam Aufgaben oder freie Zeit, z. B.: „bis Mutti Feierabend hat oder bis Papa aufsteht“ (K04§132). Zusätzlich erfolgte die Betreuung in telefonischem Kontakt. Betreuung durch die große Tochter kam nicht infrage, da das Verhältnis zwischen den beiden Schwestern angespannt ist. Wie auch in anderen Familien mit psychisch erkrankten Kindern stellten sich die Geschwisterbeziehungen als ein Problem dar (Reis et al., in diesem Heft).

Die Familie bewältigt die Krise in ihrem Kernnetzwerk. Die Beziehungen im Haushalt erweisen sich als verlässlich, werden aber extrem gefordert. Die Eltern sehen sich in der Hauptverantwortung für die Situation und nehmen diese auch an. Doch, so sagen sie, das war „schon vor der Coronazeit so …. Familie ist uns sowieso heilig, sehr heilig“ (K04§147ff).

#### Familie Krüger (K14): „Bei manchen hab’ ich Angst, dass sie sich distanzieren“^6^

Das Netzwerk der Familie Krüger zeigt Abb. 2. Mit 18 Alteri gehört es zu den größten im Sample. Die Beziehungsdichte ist dagegen besonders niedrig (0,06). Auffällig ist der hohe Anteil institutioneller Kontakte (0,5), die die Kernstruktur aus Mitgliedern der erweiterten Familie (Anteil = 0,22) als isolierte Punkte umgeben. Als „Ego“ betrachten die Befragten in diesem Fall die Kernfamilie und nicht sich selbst als Einzelperson. Daher taucht keiner der Partner in der Abbildung auf.

Herr und Frau Krüger leben gemeinsam mit ihren beiden Söhnen (10 und 13 Jahre) und den Eltern von Frau Krüger in einem Doppelhaus. Frau Krüger ist Steuerfachangestellte, Herr Krüger Industriemechaniker. Die Söhne leiden an einer genetisch bedingten geistigen Behinderung und benötigen ständige Pflege (Begleitung zur Toilette). Es fällt ihnen schwer, Routinen und Regeln zu befolgen. Aufgrund von Wutausbrüchen infolge unvorhersehbarer Ereignisse unternimmt Frau Krüger keine Ausflüge mit den beiden ohne die Begleitung ihres Mannes. Wichtigste Unterstützung im Alltag sind die im Haus lebenden Eltern von Frau Krüger.

In den ersten beiden Wochen des Lockdowns waren die Kinder zu Hause, dann in der Notbetreuung. Herr Krüger arbeitete weiter Vollzeit, Frau Krüger nur noch in Teilzeit im Homeoffice. Sie trug daher die Hauptlast der Sorgearbeit. Vor der Krise war die freie Zeit in der Woche stark durch regelmäßige Termine der beiden Söhne strukturiert (Physiotherapie, Caritas, Schwimmen, Musikschule, Arztbesuche, …), die nun alle ausfielen. Die frei gewordene Zeit musste nun von den Eltern und Großeltern ausgefüllt werden, und mit den Terminen entfielen auch die Gelegenheiten, Beratung und v. a. professionelle Unterstützung im Umgang mit den behinderten Söhnen einzuholen, wie sie z. B. in der Behinderten-Freizeiteinrichtung der Caritas angeboten wird:

Und deshalb geht er auch gerne zur Caritas, er is’ halt sehr aufdringlich und kommt sehr dicht. … Er braucht auch ma’ harte Worte …, jetz’ nich’ anschrei’n, also klare Ansagen, die mögen ihn dann trotzdem. Viele [nicht professionelle Akteur*innen] können damit nicht umgehen … andere ham Angst, dass man zu viel von ihnen erwartet. … wir sind halt schon anders, wir können nicht alles mitmachen. (K14§338)

Frau Krüger hat den Eindruck, dass einige Bekannte und Verwandte die Maßnahmen als willkommenen Anlass nehmen, den Kontakt mit der unterstützungsbedürftigen Familie herunterzufahren:

Bei manchen hab’ ich Angst, grade bei Leuten, die man besser kennt, dass sie sich distanzieren, weil sie Angst haben, dass sie so Verpflichtungen haben … dass sie gar nicht erst so in die Bredouille kommen. (K14§318)

In der Folge ist die Familie unfreiwillig auf sich selbst zurückgeworfen. In Ermangelung anderweitiger Quellen sozialer Unterstützung zeigt sich eine starke Abhängigkeit von institutionellen Akteur*innen, die wiederum kein eigenes Netz bilden, sondern einzeln angesteuert werden müssen.

#### Familie Mayer (K11): „Soll ich sagen, ich mach’ jetzt Hartzer?“

**Figure 3:**
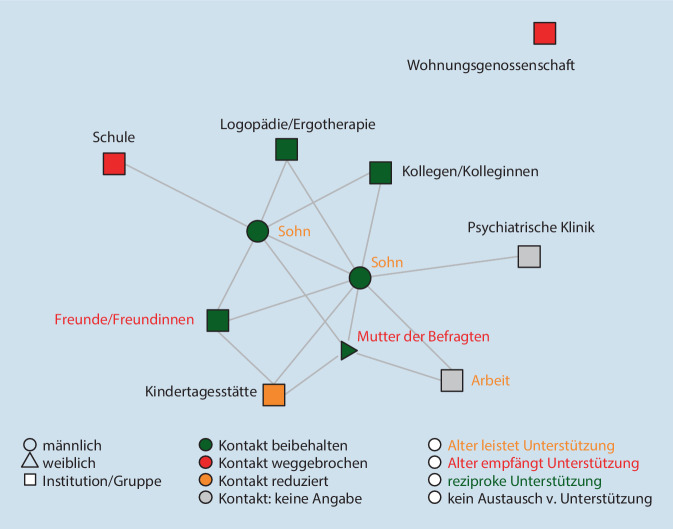

Das Netzwerk der alleinerziehenden Frau Mayer ist in Abb. 3 zu sehen. Es ist von mittlerer Größe (11) und Beziehungsdichte (0,29), verfügt aber über einen hohen Anteil institutioneller Akteur*innen (54 %). Im Zentrum stehen die beiden Söhne und die Mutter von Frau Mayer, deren Kontakt sie jedoch nur aus Pflichtgefühl aufrechterhält.

Kurz vor dem Lockdown trat sie eine Vollzeitstelle als Verkäuferin an. Um ihren neuen Job nicht zu gefährden, arbeitet sie weiter in Schichten und überlässt die Kinder in dieser Zeit sich selbst. Den kleinen Sohn könnte sie in die Notbetreuung des Kindergartens geben, tut dies aber nicht, da sich die Hol- und Bringzeiten nur schwer mit ihrer Arbeit vereinbaren lassen. Er war vor dem Lockdown zum Zweck einer eingehenden Diagnostik tagesklinisch aufgenommen worden. Während Frau Mayer arbeitet, spielen die Kinder mit der Konsole, schauen fern und essen das, was sie ihnen vorbereitet hat. Sie selbst sieht das recht unproblematisch:

Ja, L. [14-jähriger Sohn], pennt in der Regel bis 14 Uhr. Das heißt, eigentlich ist P. [6-jähriger Sohn] dann alleine. … Ja, und dann ist er halt am Gucken …, und L. ist drüben, spielt PlayStation und in der Regel sehen die sich teilweise gar nicht. … Wir ham hier auch drum rum Freunde wohnen …, die können jederzeit überall klingeln … aber bis jetzt hat nie einer was gesagt, die sind immer ganz ruhig, und es läuft wunderbar. (K11§96)

Später erzählt Frau Mayer, dass ihre Kinder, auch dann einen weitgehend entstrukturierten Alltag haben, wenn sie da ist:

Die Kinder, die waren teilweise länger wach als ich, ich bin dann abends auf der Couch eingeschlafen, weil ich müde war, weil mein Tag war anstrengend. … Der [6-jährige Sohn] hat hier echt dann teilweise bis um einse, zweie Party gemacht, und der Große sowieso …, die hatten ja überhaupt keinen Rhythmus mehr, ne, so alles an Tagesabläufen und Stabilität war ja komplett weg. (K11§108)

Sie spielt ihre Verantwortung für die Situation der Kinder unter Verweis auf die Coronalage herunter:

… ja gut, dann ist das jetzt halt so, dann isst du jetzt halt zum Frühstück Mittag, wenn du meinst, du musst das machen. Naja, dann kam auch die Entspannung zurück. (K11§108)

Es gibt mehrere Anhaltspunkte dafür, dass Akteur*innen aus dem Netzwerk die Situation kritisch hinterfragen: Kolleg*innen äußern sich irritiert, dass Frau Mayer die Kinder allein lässt. Die über eine WhatsApp-Gruppe organisierten Kita-Eltern fragen, ob der Sohn an einer Webkonferenz mit den anderen Kindern teilnehmen möchte. Die (nicht im Netzwerk aufgeführte) Kinderärztin rief an, um darauf hinzuweisen, dass der jüngere Sohn aufgrund einer Vorerkrankung zur Risikogruppe gehöre. Es scheint, als gäbe es ein Umfeld, das die Situation im Blick hat. Frau Mayer lässt sich aber nicht dreinreden. Stolz weist sie alle Anfragen und Hilfen unter Verweis auf ihre materielle Unabhängigkeit zurück:

Was soll’n wir machen? Ja klar, ich hätt’ auch sagen können, ich mach’ jetzt Hartzer, ich möcht aber nicht. … Ich mach hier nicht „just for fun, olé olé“, sondern geh’ arbeiten, ich verdien’ Geld, wir wollen leben. (K11§110)

Im Ergebnis kommt die soziale und institutionelle Unterstützung nicht bei den Kindern an. Die Problematik steigert sich in der Krise dadurch, dass die Probleme unerkannt bleiben, da sie nicht mehr regelmäßig in Institutionen wie Schule und Kita auftauchen.

## Diskussion

Soziale Netzwerke werden durch die Krise in erheblichem Maße geschwächt. Gleichzeitig sind sie die wichtigste Ressource der Krisenbewältigung. Die hier betrachteten Risikofamilien haben im Vergleich zu anderen Erhebungen (Klärner und Knabe 2016) relativ große Netzwerke, was partiell auf einen hohen Anteil institutioneller Kontakte zurückzuführen ist. Die Arten der Krisenbewältigung sind vielfältig und reichen vom „Survival in der Nische“ der Familie Fiedler (Reis 2020) über entkoppelte bzw. alleingelassene Familien (Familie Krüger) bis hin zur Gefahr der Vernachlässigung (Familie Mayer).

Wichtigste Quellen sozialer Unterstützung sind die erweiterte Familie und institutionelle Einrichtungen. Der in vielen Fällen zu beobachtende Wegfall institutioneller Unterstützung lässt sich nur unzureichend oder gar nicht kompensieren. Im schlimmsten Fall verschwinden die Kinder nicht nur aus den Einrichtungen, sondern ganz und gar aus der institutionellen und gesellschaftlichen Obhut. Ihr Wohl hängt dann allein von den Kompetenzen ihrer Eltern ab.

## Fazit für die Praxis

In Zeiten von Kontaktbeschränkungen benötigen Risikofamilien besondere Aufmerksamkeit. Es genügt nicht, darauf zu warten, dass sie von sich aus um Hilfe oder eine Notbetreuung für die Kinder bitten. Institutionen sollten proaktiv praktische und informationelle Unterstützung anbieten. Folgende Ansätze zur Verringerung der Unsicherheiten und Risiken sollten erwogen werden:

- Nutzung transparenter und niedrigschwelliger Kommunikationsstrategien (Internetseiten, Aushänge, automatischen Bandansagen, …), die die Betroffenen möglichst präzise über die aktuelle Situation aufklären: Was geht? Was nicht? Wohin kann ich mich bei Problemen wenden?
- Gewährleistung einer telefonischen oder digitalen Erreichbarkeit im Problemfall;
- Aufrechterhaltung und Priorisierung des Kontakts zu Risikofällen im Sinne der Prävention, z. B. durch Anrufe, Hausbesuche und Einladungen zum Gespräch.

## Footnotes

1 In einem der 19 Fälle ließ sich die Netzwerkerhebung im Anschluss an das qualitative Interview nicht realisieren.

2 Bei den zu 100 % fehlenden Nennungen handelt es sich um Alteri, die außerhalb der Namensgeneratorabfrage benannt wurden, z. B., wenn den Interviewten im abschließenden Gespräch über das Netzwerk noch eine weitere Person eingefallen ist.

3 Die Namen aller Familien wurden geändert.

4 Namen geändert.

5 Die Fallskizze entstand auf Basis eines Portraits von Elisabeth Zessin, die das Interview im Rahmen eines forschungspraktischen Seminars führte (Reis et al., in diesem Heft).

6 Die Fallskizze entstand auf Basis eines Portraits von Mareike Ledigen, die das Interview im Rahmen eines forschungspraktischen Seminars führte (Reis et al., in diesem Heft).
